# A Case-Control Study on the p73 G4A Gene Polymorphism and Susceptibility to Breast Cancer in an Iranian Population

**Published:** 2019-10

**Authors:** Zeinab TAVAKKOL AFSHARI, Zahra GHOLIZADEH, Amin Reza NIKPOOR, Jalil TAVAKKOL AFSHARI, Rashin GANJALI, Fatemeh HOMAEI SHANDIZ, Khadijeh JAMIALAHMADI

**Affiliations:** 1. Department of Immunogenetic and Cell Culture, Immunology Research Center, School of Medicine, Mashhad University of Medical Sciences, Mashhad, Iran; 2. Department of Allergy and Immunology, School of Medicine, Mashhad University of Medical Sciences, Mashhad, Iran; 3. Molecular Medicine Research Center, Hormozgan Health Institute, Hormozgan University of Medical Sciences, Bandar Abbas, Iran; 4. Cancer Research Center, Mashhad University of Medical Sciences, Mashhad, Iran; 5. Biotechnology Research Center, Pharmaceutical Technology Institute, Mashhad University of Medical Sciences, Mashhad, Iran; 6. Department of Medical Biotechnology and Nanotechnology, School of Medicine, Mashhad University of Medical Sciences, Mashhad, Iran

**Keywords:** Tumor protein p73, Polymorphism, Breast cancer

## Abstract

**Background:**

The tumor protein p73 (TP73) is a homolog of TP53 family. Ectopic p73 overexpression largely mimics p53 activities as a tumor suppressor and activates the transcription of p53-responsive genes and as a result induce apoptosis. This study aimed to investigate the association between p73 G4A polymorphism and the risk of breast cancer in a northeastern Iranian population.

**Methods:**

This case-control study was performed on 105 patients who admitted in educational hospitals of Mashhad University of Medical Sciences, Iran during 2013–2015, with breast cancer as case group and 120 healthy women as the control group. PCR-CTPP method was used to investigate the relationship between the p73 G4A polymorphism and the risk of breast cancer.

**Results:**

There was no significant association between the AA genotype of the p73 G4A polymorphism and breast cancer in case and control groups. Although G allele frequency was higher in the case group, the abundance of this allele between case and control groups was not statistically meaningful and, as a result, not associated with the risk of breast cancer in this study group.

**Conclusion:**

There was no association between G4A p73 polymorphism and the risk of breast cancer in a northeastern Iranian population.

## Introduction

Cancer is the second leading cause of death worldwide after cardiovascular disease (1). Breast cancer (BC) is the most commonly diagnosed cancer in women in the world (2). Women in menopausal phase are at higher risk of this cancer (3). On average, every 19 second one woman is diagnosed with breast cancer and one person per minute dies from this disease (4). Global statistics show the annual incidence of BC is increasing and occurring more rapidly in countries with a low incidence rate of this cancer (2, 5). Breast cancer occurs more frequently in developed countries (6), due to higher prevalence of BC risk factors, such as older age at first pregnancy, low parity, high-calorie intake, sedentary occupation and use of hormonal replacement therapy (7, 8). This cancer also is becoming an issue for public health in Iran. It is the third cause of cancer death in Iran after coronary heart disease and accidents (9). In Iran, breast cancer ranks first among cancers diagnosed in women; comprising 24.4% of all malignancies with a crude incidence rate and age-standardized rate (ASR) of 17.4 and 23.1 per 100,000, respectively (10). Moreover, even after adjusting for age, Iranian young women are at relatively higher risk for developing BC than their Western counterparts (11).

Tumor protein 73 (TP73) is a homolog of p53, coded via a polymorphic gene in 1p36.3 region (12). p73 is one of the well-known tumor suppressor genes imprinted in some human tissues. When this protein is overproduced, it can activate transcription of responsive genes for p53 that are important in cell cycle control, DNA repair and apoptosis. Furthermore, this protein prevents cell growth through apoptosis induction in a similar way to p53 (13). The p53 gene as a tumor suppressor and its homolog, p73, play a very important role in human malignancies (12).

Up to now, at least 19 polymorphisms have been recognized in p73. Ten polymorphisms are in exon region, eight in intron region and one is in the region of gene promoter. However, functionality of most of these polymorphisms has not been recognized yet (14). The 1p36.3 region, which is coding for the p73 protein, is deleted in different malignancies via loss of heterogeneity. Although mutation in p73 gene is rare and occurs in less than 2% of all cancers, the lack of heterogeneity in locus p73 is common in a variable rate in different cancers (15). As a result, the genetic changes of p73 gene are critical in malignancies (13). Two related single nucleotide polymorphisms (SNPs) in regions 4 (G>A) and 14 (C>T) of exon 2 in p73 gene result in formation of a stem-loop structure that can affect gene function via changes in its expression. Molecular epidemiology studies have indicated the association between this polymorphism and the risk of cancers in human, however, the results are inconsistent (15).

The aim of this study was to investigate the relation between G4A polymorphism in p73 gene and the risk of breast cancer in northeast population of Iran.

## Materials and Methods

### Study population

This case-control study was performed on 105 clinically and pathologically confirmed breast cancer patients who admitted in educational hospitals of Mashhad University of Medical Sciences, Iran during 2013–2015 as case group and 120 age-matched healthy women as control group were enrolled.

All participants were asked to complete the consent form, approved by the Institutional Ethical Committee and Research Advisory Committee of Mashhad University of Medical Sciences, Mashhad, Iran.

Demographic variables such as age, menopause status, family history of breast cancer, and marriage status were obtained from all participants.

### Genotyping

Five mililiter of venous blood was taken from each case study and put in test tubes containing EDTA. Genomic DNA of leukocytes was extracted using commercially available kit (Biogene Company, IRAN) according to the manufacturer's protocol. Genotyping of p73 G>A was determined by polymerase chain reaction with confronting two-pair primers (PCR-CTPP), modified from a technique (13). The region of exon 2 of the p73 gene was amplified by PCR method and was multiplied through two pairs of specific primers for the two alleles (Table 1).

**Table 1:** Primer sequences and specification of amplified fragments for p73 G4A genotyping

***Primer***	***Primer sequence***	***Length of fragments***	***Genotyping***
F1	5'-CCA CGG ATG GGT CTG ATC C-3'	270bp	Wild type G allele
R1	5'-GGC CTC CAA GGG CAG CTT-3'		
F2	5'-CCT TCC TTC CTG CAG AGC G-3'	193bp	Mutant type A allele
R2	5'-TTA GCC CAG CGA AGG TGG-3'		

PCR amplifications for both of primer pairs were performed in a 20 μl reaction volume containing 2 μL buffer, 50 μM dNTPs, 2.5 μM MgCl2, 0.5 unit of Taq polymerase with 100ng of genomic DNA. The amplifications were done as follows: initial denaturation at 95°C for 5 min followed by 35 cycles of denaturation at 95°C for 1 min, annealing at 66 °C for 45 sec, DNA extension at 72°C for 2 min and final extension at 72°C for 5 min.

All amplification cycles were performed in Biometra Thermal cycler (Biometra Ltd., Kent, UK). The PCR products were separated by electrophoresis on 2% agarose gels containing ethidium bromide and visualized by UV light. The product size of G and A alleles was shown in Table 1. For quality control, a random of 10% of the samples were repeated, and the results were 100% concordant.

### Statistical analysis

Case and control groups were compared according to the difference in their genotype frequency via chi-square test using SPSS software version 17.0 software (Chicago, IL, USA). The amount of genetic divergence within population was tested via Hardy-Weinberg. Moreover, Independent t-test, logistic regression test was used and *P*-value less than 0.05 were considered statistically significant.

## Results

The present study was performed on 105 women with breast cancer with average age of 46.1±10.2 yr and 120 healthy control subjects as a control group with an average age of 43.8±11.8 yr old (*P*=0.07). The frequency of genotype between case and control groups was as shown in Table 2.

**Table 2:** Genotype and allele frequencies of G4A p73 polymorphism in patients with breast cancer compared to the control group

***Variable***		***Patients (n=105)(%)***	***Controls (n=120)(%)***	**P-*value***	***OR (CI 95%)***
p73 Genotype	G/G	77(73.3)	79(65.8)	0.36	1.00 (Reference)
	A/G	25(23.8)	34(28.3)		0.75 (0.41– 1.38)
Co dominant	A/A	3 (2.9)	7(5.8)		0.44 (0.11– 1.63)
	HWE p	0.58	0.20		
Dominant	G/G	77(73.3)	79(65.8)	0.22	1.00 (Reference)
	A/G- A/A	28(26.7)	41(34.2)		0.70 (0.39–1.24)
Recessive	G/G- A/G	102 (97.1)	113(94.2)	0.27	1.00 (Reference)
	A/A	3(2.9)	7(5.8%)		0.47(0.12– 1.88)
Over dominant	G/G-A/A	80(76.2)	86(71.7)	0.44	1.00 (Reference)
	A/G	25(23.8)	34(28.3)		0.79(0.43–1.44)
p73 Alleles	G	179(85)	192(80)	0.17	1.00 (Reference)
	A	31(.15)	48(20)		0.96 (0.42– 1.13)

The genotype frequency of p73 gene G4A polymorphism does not show any significant association with the risk of breast cancer in either patient or control groups (*P*=0.17). Moreover, there was no association between the different models of G4A p73 polymorphism such as dominant, recessive, over dominant and codominant and increased risk of breast cancer (Table 2). The genotype distributions were in Hardy-Weinberg equilibrium in controls and patients group (X^2^ *P*-value for patient: 0.58 and control: 0.20).

To examine the association between average age of patients in time of diagnosis of breast cancer and the G4A p73 polymorphism, the patients were divided into two groups; below and above than 35 yr according to their age of diagnosis. The frequency of genotypes between age-classified patients was not statistically significant (*P*=0.589). The highest and lowest average age of patients with cancer was observed in genotype GG and AA, respectively (Table 3).

**Table 3:** G4A p73 polymorphism genotype frequencies among patients based on classified age of cancer diagnosis

***Categorized age***		***Below 35 years (%)***	***Above 35 years (%)***	***Total (%)***	**P*-value***
p73 genotypes	G/G (%)	12(15.6)	65 (84.4)	77(100)	
	A/G (%)	5(20)	20 (80)	25(100)	0.589
	A/A (%)	0 (0)	3(100)	3(100)	
	Total(%)	17 (16.2)	88 (83.8)	105 (100)	

Furthermore, pathological type of breast cancer stratified analysis in patients group were shown in Table 4. In stratified analysis, no statistically significant association was observed between different pathological types of breast cancer and the inheritance of p73 G4A genotypes (*P*= 0.811).

**Table 4:** Association between G4A p73 SNP genotypes frequencies and pathologically diagnosis of breast cancer

***P73 genotype***	***G/G (%)***	***A/G (%)***	***A/A (%)***	***Total (%)***	**P*-value***
Invasive ductal carcinoma	60 (78)	18 (75)	3 (100)	81 (77.9)	
Medullar Carcinoma	5(6)	2(6.2)	(0)	7(5.9)	
Comedo carcinoma	3(4)	3(12.5)	(0)	6(5.9)	
Invasive lobularcarcinoma	8(10)	0(0)	(0)	8(7.4)	
Musinous carcinoma	1(2)	2(6.2)	(0)	3(2.9)	0.811
Total	77 (100)	25 (100)	3(100)	105 (100)	

As shown in Table 4, the most and least frequent type of breast cancer among patients were invasive ductal carcinoma and mucinous carcinoma (77.9% and 2.9% of total patients, respectively). Finally, patients based on their menopausal status were divided into two groups (premenopausal and post-menopausal) and their relationship with p73 G4A genotypes was examined and our findings showed no significant association between mentioned polymorphism and menopause status (*P*=0.31) among our patients' participants.

## Discussion

In this study, there was no statistically significant association between G4A polymorphism in exon 2 of the p73 gene and the risk of breast cancer in our study population. Moreover, although the G mutant allele frequency was higher in breast cancer patients than in controls, this difference was not statistically meaningful.

p73 is a p53 homolog that has a similar structure and function to it. p73 can activate the transcription of p53-responsive genes and plays an essential role in cell cycle events and DNA damage mechanisms. Although the role of p73 G4C14>A4T14 polymorphism and the risk of cancer has been investigated for different types of cancers in populations with various genetic and social backgrounds (16), to date inconsistent data are available (13, 15, 17–20). We examined the association of p73 G4C14>A4T14 polymorphism and susceptibility to breast cancer in a series of 105 breast cancer cases and 120 age-matched population control participants as a sample of Iranian population. Studies showed that a genotype AT/AT or AT allele can lower the risk of advanced esophageal cancer in Irish population and lung cancer in Chinese people. On the other hand, AT allele can enhance the risk of susceptibility to some type of cancers such as esophageal, cervical and endometrial in Japanese population and lung cancer in a non-Hispanic white population (16). Moreover, there are many studies that reported no association between mentioned polymorphism and the risk of cancer (16).

In contradiction to some studies, our study showed that there was no meaningful association between p73 G4A polymorphism and the risk of breast cancer. In a study on Chinese population, there was a significant association between GC/GC genotype and the weak prognosis of breast cancer. Furthermore, cases with GC/GC genotype had a higher risk of advanced breast cancer compared to those with either GC/AT or AT/AT genotype (21). An epidemiologic study was performed on association between this polymorphism and the risk of endometrial cancer in Japanese population. There was a strong correlation between p73g4a polymorphism and type I tumor while there was not any significant association between this polymorphism and endometrial cancer type II (13). These results are in consistency with our results which may be because of the limited case studies. Furthermore, ethology of genetic polymorphisms between type I and II are different. In our study, highest and lowest genotypes were GG with 73.3% and AA with 2.9%, respectively which was consistent with the results reported on both types of endometrial cancer (13).

The impact of different genotypes of p73g4a polymorphism on tumor development can be explained by different functions of p73 protein in various tissues and tumors. Some SNP of p73 are identified but none of them can be substituted by amino acids in proteins (22, 23). G4A p73 polymorphism can affect translation functionality and overexpression of p73 protein. This protein is similar to p53 in attaching to DNA, domain oligomerization and functionality (13). An increase of p73 protein expression was observed in some tumors with defective p53. This increased expression is in format of a slight compensation mechanism due to the lack of p53 functionality (24). If this compensation due to decreased expression of p53 protein is insufficient, it can effect on pathogenesis of some types of cancers (13).

## Conclusion

There was no statistically significant association between G4A polymorphism in exon 2 of the p73 gene and the risk of breast cancer in a sample of northeast Iranian population.

## Ethical considerations

Ethical issues (Including plagiarism, informed consent, misconduct, data fabrication and/or falsification, double publication and/or submission, redundancy, etc.) have been completely observed by the authors.
